# Evaluating malaria prevalence and land cover across varying transmission intensity in Tanzania using a cross-sectional survey of school-aged children

**DOI:** 10.1186/s12936-022-04107-8

**Published:** 2022-03-09

**Authors:** Cedar L. Mitchell, Billy Ngasala, Mark M. Janko, Frank Chacky, Jessie K. Edwards, Brian W. Pence, Ally Mohamed, Lwidiko E. Mhamilawa, Twilumba Makene, Thwai Kyaw, Fabrizio Molteni, Humphrey Mkali, Ssanyu Nyinondi, Bilali Kabula, Naomi Serbantez, Erin L. Eckert, Chonge Kitojo, Erik Reaves, Michael Emch, Jonathan J. Juliano

**Affiliations:** 1 Department of Epidemiology, Gillings School of Global Public Health, University of North Carolina, Chapel Hill, NC USA; 2 Muhimbili University of Health and Allied Sciences, Dar es Salam, Tanzania; 3 Institute for Health Metrics and Evaluation, University of Washington, Washington, USA; 4 Gender, Elderly and Children, Ministry of Health, Community Development, Dodoma, Tanzania; 5 National Malaria Control Programme, Dodoma, Tanzania; 6 Division of Infectious Diseases, University of North Carolina School of Medicine, Chapel Hill, NC USA; 7 Tropical and Public Health Institute, Basel, Switzerland; 8 RTI International, Dar es Salaam, Tanzania; 9 US President’s Malaria Initiative (PMI), United States Agency for International Development, Dar es Salaam, Tanzania; 10 RTI International, Washington, DC USA; 11 President’s Malaria Initiative, U.S. Centers for Disease Control and Prevention, Dar es Salaam, Tanzania; 12 Department of Geography, University of North Carolina, Chapel Hill, NC USA

**Keywords:** Land cover, Transmission intensity, Malaria

## Abstract

**Background:**

Transmission of malaria in sub-Saharan Africa has become increasingly stratified following decades of malaria control interventions. The extent to which environmental and land cover risk factors for malaria may differ across distinct strata of transmission intensity is not well known and could provide actionable targets to maximize the success of malaria control efforts.

**Methods:**

This study used cross-sectional malaria survey data from a nationally representative cohort of school-aged children in Tanzania, and satellite-derived measures for environmental features and land cover. Hierarchical logistic regression models were applied to evaluate associations between land cover and malaria prevalence within three distinct strata of transmission intensity: low and unstable, moderate and seasonal, and high and perennial.

**Results:**

In areas with low malaria transmission, each 10-percentage point increase in cropland cover was associated with an increase in malaria prevalence odds of 2.44 (95% UI: 1.27, 5.11). However, at moderate and higher levels of transmission intensity, no association between cropland cover and malaria prevalence was detected. Small associations were observed between greater grassland cover and greater malaria prevalence in high intensity settings (prevalence odds ratio (POR): 1.10, 95% UI: 1.00, 1.21), and between greater forest cover and reduced malaria prevalence in low transmission areas (POR: 0.74, 95% UI: 0.51, 1.03), however the uncertainty intervals of both estimates included the null.

**Conclusions:**

The intensity of malaria transmission appears to modify relationships between land cover and malaria prevalence among school-aged children in Tanzania. In particular, greater cropland cover was positively associated with increased malaria prevalence in areas with low transmission intensity and presents an actionable target for environmental vector control interventions to complement current malaria control activities. As areas are nearing malaria elimination, it is important to re-evaluate environmental risk factors and employ appropriate interventions to effectively address low-level malaria transmission.

**Supplementary Information:**

The online version contains supplementary material available at 10.1186/s12936-022-04107-8.

## Background

Decades of malaria control interventions and targeted treatment have led to reduced malaria prevalence in many areas, but other areas remain at high risk of the disease. As malaria transmission patterns are changing, stratification of infection risk is becoming more apparent [1–3]. In sub-Saharan Africa, since 2010, the largest transition in malaria transmission dynamics has occurred among high transmission intensity settings (*Plasmodium falciparum* parasite rate > 50%) downshifting to moderate intensity (*P. falciparum* parasite rate 10–50%) [1]. Changing levels of transmission intensity disrupt established malaria ecological dynamics and may result in different relationships between ecological covariates and malaria across different levels of transmission. Potential modification of the environmental predictors of malaria by transmission intensity could inform malaria control programmes of differing intervention needs of communities based on underlying transmission profiles and could further optimize allocation of resources for the control of malaria.

A complex network of factors drives malaria transmission. Ecological factors, such as climate, land cover and land use, and human interventions interact to shape the dynamics of when, where and how frequently malaria infections occur [3]. Profiles of temperature, precipitation, elevation, and land cover have long been used to model and predict the occurrence of malaria in endemic areas and highlight locations with elevated risk of the disease to prioritize interventions [1–5]. Human modification of land cover, particularly for agriculture, has been found to be associated with increased malaria prevalence among children in the Democratic Republic of the Congo (DRC) [6], Malawi [7], Tanzania [8–10], Kenya, Burundi, and even re-establishment of malaria in Madagascar [11, 12]. As populations expand within sub-Saharan Africa, modification of current land cover to develop agricultural areas is also expanding [13]. If relationships between land cover and malaria prevalence depend on transmission intensity, it is important to understand how different classes of land cover may affect malaria dynamics under changing levels of transmission intensity.

In this study, relationships were evaluated between land cover and malaria prevalence stratified by transmission intensity in the United Republic of Tanzania. An estimated 93% of the mainland Tanzanian population remains at risk of malaria [14], however malaria risk stratification across the country is highly heterogenous [15, 16]. Recently, Tanzania has transitioned from a highly endemic setting to moderate transmission intensity, driven in part by many urban areas shifting to very low or no transmission settings. However, numerous rural areas remain at high levels of transmission [16]. The widening gaps in intensity of malaria transmission in Tanzania offer a unique opportunity to explore differences in ecological conditions known to influence malaria epidemiology, notably land cover and land use, between areas under different transmission intensities.

This study utilized data from a parasitological survey of school-aged children in mainland Tanzania to characterize environmental and land cover features of differing levels of transmission intensity, and to test the hypothesis that the strength and directionality of relationships between land cover and malaria prevalence might differ across low, moderate, and high levels of transmission intensity.

## Methods

### Study setting

Tanzania is situated in the East African highlands with a sloping coastal zone along the Indian Ocean. The climate is considered tropical, however high mountainous regions and sweeping arid regions of the highlands exhibit temperate climates and are less suitable for malaria transmission [14, 17]. Two distinct rainfall patterns occur across Tanzania: the north and east experience two rainy seasons, short rains from October to December and long rains March through May; southern, western and central areas experience one longer wet season from October through to April or May [17]. Differing patterns of rainfall contribute to varying levels of malaria vector abundance, transmission intensity and seasonality resulting in perennial malaria transmission across 60% of endemic areas, stable seasonal transmission in another 20%, and unstable seasonal, low intensity transmission in the remaining 20% of areas [14]. Land use in Tanzania is dominated by the agricultural sector, which covered 45% of the total land area in 2014 (13 million hectares) [18]. Much of the remaining land cover includes protected savannah and grasslands, primary forest, and water bodies.

### Study design

This study utilized data from the 2017 Tanzania School Malaria Parasitological Survey (SMPS), which was administered to school pupils aged 5–16 years old from July to November, 2017 on mainland Tanzania. The Tanzania SMPS was cross-sectional and followed a multi-stage sampling design that was geographically representative (stratified by elevation) across eight of the 26 Tanzania regions. The eight selected regions were chosen to represent heterogenous malaria transmission levels and were surveyed during their respective rainy season. Within each region, schools were randomly selected, the number of schools selected was proportional to the regional population, and within each school 100 pupils were randomly sampled. Sampled pupils were interviewed to collect a discrete set of demographic and health variables and were asked to provide a dried blood spot (DBS) sample for malaria testing. Interviews and DBS samples were obtained for all sampled students at each school who provided informed consent; no other exclusion criteria were imposed. DBS samples were transported to the University of North Carolina for molecular detection of falciparum malaria via PCR amplification of the lactate dehydrogenase gene as described previously [19]. Individual falciparum malaria infection as measured by PCR is the main outcome for this study.

Land cover is the main exposure for this analysis and was derived from the European Space Agency Climate Change Initiative (CCI) land cover data product, which measures and classifies global land cover annually. Land cover was re-grouped into four classes based on the CCI land cover classification system [20] as cropland, forest, grassland, or flooded/swamp land. For each school, land cover values within a 10-km buffer were extracted and the per cent of each land cover class estimated by summing the number of pixels for each land cover class, dividing by the total number of pixels extracted for each school, and multiplying by 100. Land cover was coded as a continuous variable to reflect the reality of fragmented landscapes and avoid misclassification bias in majority land cover-based approaches. Additionally, a 10-km buffer was used because it corresponds to the maximum flight distance of a blood-fed female *Anopheles spp.* mosquito [21], and also captures short-distance, daily human activity patterns in and around a village, including travel between home and school for the surveyed children.

Demographic and environmental covariates were extracted from the SMPS, remote sensed satellite platforms, and other geographic datasets. From the SMPS, pupil age, gender, and school malaria transmission intensity were extracted. Transmission intensity was estimated from the 2014–2015 Tanzania SMPS as the ratio of *Plasmodium falciparum* positive rapid diagnostic tests to the total number of rapid diagnostic tests administered during the study [15, 16]. Environmental covariates were extracted by school location and included precipitation, temperature, vegetation, elevation, population density, and proximity to water bodies. Measures of precipitation were obtained through the Climate Hazards Group InfraRed Precipitation with Station data (CHIRPS). Temperature and vegetation were derived from the Moderate Resolution Imaging Spectroradiometer satellite platform; temperature was measured in degrees above 16 °C, the minimum temperature for *P. falciparum* development [22, 23], and vegetation was measured using the Enhanced Vegetation Index (EVI) scale, which ranges from 0.1 to 1 with higher numbers indicating greater abundance and richness of vegetation [24]. Precipitation, temperature and vegetation were averaged monthly with temperature lagged one month prior to the month of interview to account for temporal delays in the *Plasmodium* life cycle [25, 26]. Elevation was extracted from the Shuttle Radar Topography Mission, population density was derived from the 2017 WorldPop gridded population density raster, and proximity to water was measured as metres from each school GPS location to the closest river or body of water.

### Stratification by transmission intensity

Sub-national stratification of malaria risk is important for contextualizing expected impacts of malaria control interventions [5, 27] and is often used by national malaria control programmes to define operational units for allocation of resources and to direct control policies [28, 29]. Thus, to evaluate whether associations between land cover and malaria prevalence differ between strata of transmission intensity, all analyses were stratified by 3 levels of transmission intensity: low, moderate and high. Low transmission intensity corresponded to an estimated parasite prevalence of 0–10%, moderate to a parasite prevalence of 11–50%, and high to a parasite prevalence of > 50%. Categorization of transmission intensity was adapted from previously published classifications of malaria endemicity among many sub-Saharan African countries [1, 2, 30], including Tanzania [16, 31], as hypo-endemic (low), meso-endemic (moderate), and hyper-holo-endemic (high) and retain the same prevalence intervals [31]. Measures of parasite prevalence were based on results from the 2014–15 Tanzania SMPS.

### Statistical analysis

The extent to which *P. falciparum* infection varied across the three levels of transmission intensity and by individual and school-level variables were first evaluated. Individual-level variables included age and gender, and school-level variables included per cent cropland, per cent forest, per cent grassland, per cent flooded/swamp land, per cent other land (urban or bare), temperature, precipitation, vegetation, elevation, population density, and proximity to water (presented in Table 1). *Plasmodium falciparum* infection was dichotomized as positive or negative for each pupil; at the school level, negative *P. falciparum* status indicated no infections were detected at the school, while positive status reflected one or more infections detected. Median values and interquartile ranges (IQR) were estimated for all continuous variables.

**Table 1:** Demographic and environmental features of the study population stratified by *Plasmodium falciparum* PCR status and by transmission intensity

	Overall	Transmission intensity					
		Low		Moderate		High	
		*P.f.* neg	*P.f.* pos	*P.f.* neg	*P.f.* pos	*P.f.* neg	*P.f.* pos
*Individual level*							
n	17,131	6,076	69	1,829	351	5,938	2,868
Age (years)	11 [9, 13]	11 [9, 12]	12 [10, 14]	11 [9, 13]	12 [10, 14]	11 [9, 13]	12 [10, 13]
Male (%)	8,457 (49)	3,020 (50)	33 (48)	895 (49)	190 (54)	2,761 (46)	1,558 (54)
*School level*							
n	182	41	20	5	23	2	91
Per cent cropland	14 [3, 32]	3 [1, 14]	9 [3, 15]	39 [28, 39]	16 [7, 44]	47 [44, 50]	23 [9, 40]
Per cent forest	16 [5, 36]	33 [9, 43]	23 [11, 41]	3 [1, 22]	11 [3, 18]	12 [7, 18]	11 [3, 26]
Per cent grassland	49 [27, 68]	54 [45, 66]	51 [36, 60]	15 [9, 36]	40 [18, 78]	21 [21, 22]	46 [23, 68]
Per cent flooded/ swamp	0 [0, 5]	0 [0, 1]	0 [0, 5]	37 [6, 42]	1 [0, 25]	8 [4, 11]	0 [0, 9]
Per cent other land cover^a^	0 [0, 1]	0 [0, 3]	0 [0, 1]	0 [0, 3]	0 [0, 0]	12 [6, 18]	0 [0, 0]
Temperature (> 16 °C)	16 [11, 19]	13 [9, 18]	14 [10, 17]	13 [13, 14]	15 [11, 19]	17 [17]	18 [14, 19]
Precipitation	1 [1, 2]	1 [1, 2]	1 [1]	2 [1, 2]	1 [1]	3 [2, 3]	2 [1, 3]
Vegetation	0.22 [0.18, 0.31]	0.21 [0.17, 0.28]	0.22 [0.18, 0.27]	0.13 [0.12, 0.25]	0.20 [0.15, 0.40]	0.26 [0.23, 0.29]	0.25 [0.19, 0.32]
Elevation (m)	1208 [973, 1443]	1505 [1282, 1650]	1460 [1169, 1619]	1133 [553, 1177]	1058 [544, 1344]	1228 [1212, 1244]	1177 [533, 1339]
Population density (per sq km)	102 [55, 209]	147 [52, 502]	95 [64, 290]	114 [90, 838]	72 [48, 202]	317 [177, 456]	104 [63, 170]
Proximity to water (km)	3 [2, 6]	3 [2, 6]	3 [1, 7]	1 [1, 2]	3 [1, 4]	6 [4, 7]	4 [2, 6]

Data are n (%), or median, [IQR]. *P.f.: Plasmodium falciparum*. Land cover percentages are presented as median values for each stratum and are not expected to sum to 100%

^a^ Other land cover classes include urban and bare

Hierarchical logistic regression models were then used to evaluate associations between each land cover class and malaria prevalence odds while accounting for the multi-level structure of the SMPS data and covariates. All models were implemented in a Bayesian framework using integrated nested Laplace approximation methods [32]. A random intercept was included to adjust for correlation between pupils within schools. For each of the three land cover classes (cropland, forest, grassland), crude associations and associations adjusted for confounding variables were estimated, which included age and gender (individual level), and temperature, precipitation, vegetation, elevation, population density, and proximity to a body of water (school level) within each stratum of transmission intensity for a total of 18 malaria prevalence odds ratio estimates. These variables were selected based on findings from previous studies [2, 4, 16, 22, 26] and were evaluated for their potential to confound relationships between land cover and malaria prevalence using a directed acyclic graph analysis. Additionally, possible residual spatial confounding was explored in the relationship between land cover and malaria prevalence across schools by adding a spatially varying intercept. Model fit was compared using deviance information criteria with the best fitting model having the smallest DIC by a margin of 3 points or more [33]. All models were run using the ‘INLA’ package in R version 4.0.4 [32].

Each land cover class (cropland, forest, grassland) was individually coded as continuous and scaled by 10, thus each regression coefficient corresponds to the difference in log prevalence odds of malaria corresponding to a 10-percentage point difference in land cover. Relationships between land cover and malaria prevalence were assumed to be linear. All other continuous variables (age, temperature, precipitation, vegetation, elevation, population density, proximity to a water body) were mean centred and modelled linearly. Gender was the only categorical variable and was coded as male or female. Any observations with missing data were excluded from the analysis (n = 6).

### Ethical approval

Approval for this study was obtained from the University of North Carolina Institutional Review Board and from the Tanzania National Institute for Medical Research.

## Results

**Fig. 1:**
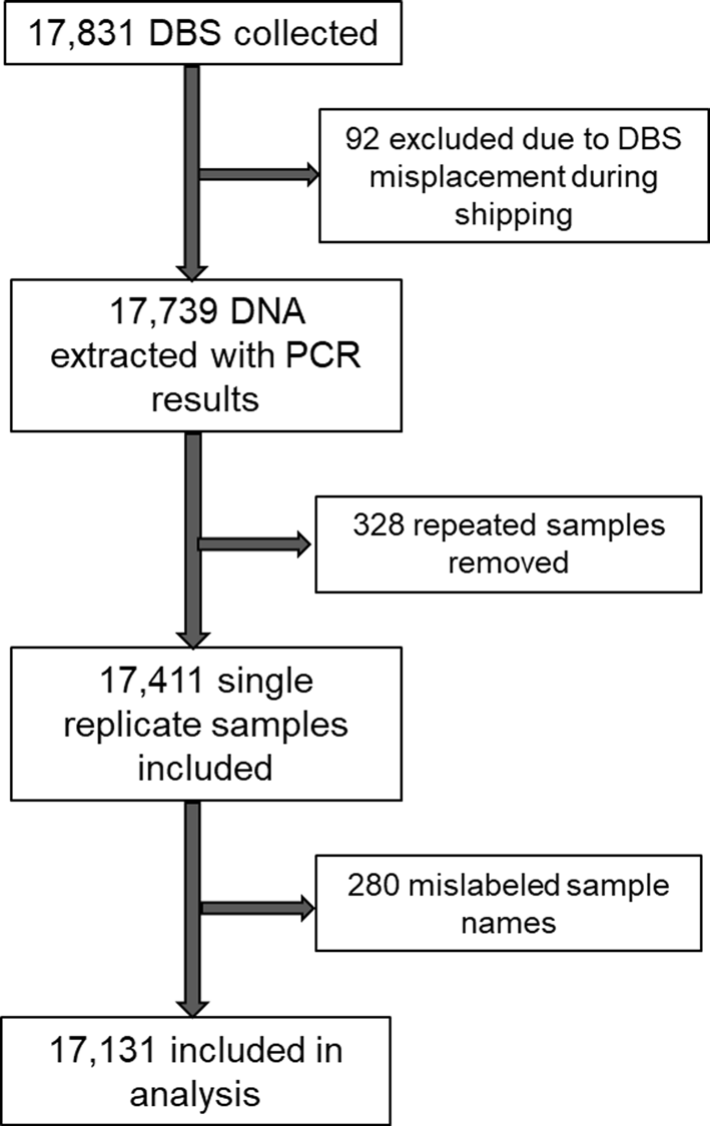
Selection of participants from the 2017 School Malaria Parasitological Survey into final analysis cohort

**Fig. 2:**
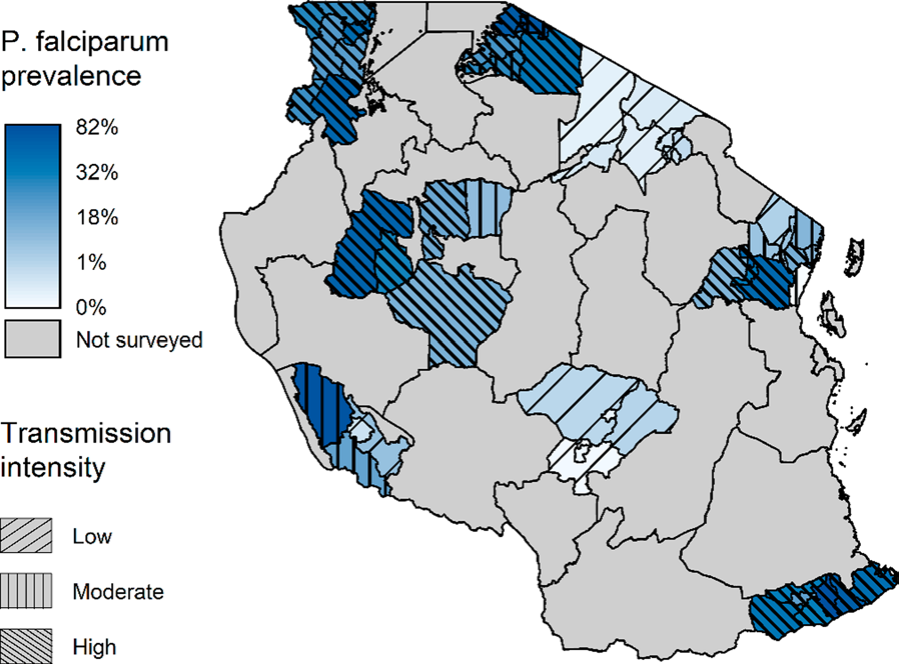
Estimated falciparum malaria per cent prevalence aggregated at council level based on the 2017 School Malaria Parasitological Survey results. Transmission intensity overlays prevalence colouring, changing directionality and density of hatching represents different transmission intensities

A total of 17,131 pupils across 182 schools within 59 district councils in eight regions had available falciparum malaria PCR results and could be linked with SMPS metadata for downstream analyses (Fig. 1). The overall prevalence of malaria in the study population was 19.2% with a 95% uncertainty interval (UI) of 18.6, 19.8%. By strata of transmission intensity, the prevalence of malaria was 1.1% (95% UI: 0.9, 1.4%) in low intensity areas, 16.1% (95% UI: 14.6, 17.7%) in moderate intensity areas, and 32.6% (95% UI: 31.6, 33.6%) in high transmission intensity areas. Across the 59 district councils included in the study, malaria prevalence ranged from 0% among several councils in Arusha, Iringa and Tanga regions to 82% in Newala Council of Mtwara (Fig. 2, Additional file 1: Table S1).

Demographic, environmental and land cover features of the study population stratified by *P. falciparum* positivity and transmission intensity are presented in Table 1. Overall, the median age of pupils was 11 years (IQR: 9–13), and 49% were male. Among malaria-positive pupils in moderate and high transmission intensity areas, the median age was slightly higher and a higher percentage were male compared with *P. falciparum-*negative pupils in these strata. The percentage of schools with any malaria-positive pupils was highest among high-transmission intensity schools at 98% (91/93), followed by 82% (23/28) of schools in moderate-transmission areas, and at low transmission intensity, 33% (20/61) of schools were positive. Cropland was more abundant among moderate- and high-transmission intensity schools. Forest and grassland accounted for higher median percentages of land cover in low-transmission intensity areas. Flooded areas and swamps covered very little land. Median temperature was higher among malaria-positive schools and increased with transmission. Median precipitation and vegetation values were low overall and changed little by malaria and transmission intensity. Median elevation values were lower among *P. falciparum*-positive schools compared with negative schools and decreased with increasing transmission intensity; the IQR ranges, however, were large. Population density was consistently lower among malaria-positive schools compared with malaria-negative schools at all levels of transmission intensity. The median distance to a river or body of water for all schools was 3 km and varied little by malaria status and transmission intensity.

### Analysis of associations between land cover and malaria prevalence

Due to the low percentage of land cover by flooded/swamp lands, the only modelled associations were for cropland, grassland, and forest land cover. Odds ratios were estimated for a 10-unit difference in each land cover class alone (crude) and adjusted for a set of confounding variables which included age, gender, temperature, precipitation, vegetation, elevation, population density, and proximity to water within each stratum of transmission intensity. Estimated malaria prevalence odds ratios from these models are presented in Table 2; additional results from the adjusted models for covariate beta estimates and 95% UIs are available in Additional file 1: Table S2. For all land cover classes, model fit statistics were similar between non-spatial models and models that included a spatially varying intercept. Because the fit of the more complex spatial models was not favoured over the simpler non-spatial models, it was assumed that any confounding due to space was minimal at the spatial scales evaluated and outcomes from the non-spatial models were reported. A comparison of results from the non-spatial and spatial modelling approaches can be found in Additional file 1: Table S3. Posterior malaria prevalence odds ratio estimates for the three land cover classes by transmission intensity are described below.

**Table 2:** Crude and adjusted posterior malaria prevalence odds ratios and 95% uncertainty intervals for each of the three main land cover classes stratified by transmission intensity

	Low transmission		Moderate transmission		High transmission	
	Crude OR (95% UI)	Adjusted OR (95% UI)	Crude OR (95% UI)	Adjusted OR (95% UI)	Crude OR (95% UI)	Adjusted OR (95% UI)
Cropland	2.00 (1.19, 3.47)	2.44 (1.27, 5.11)	0.89 (0.61, 1.31)	0.87 (0.60, 1.24)	0.95 (0.85, 1.06)	0.94 (0.84, 1.06)
Grassland	0.95 (0.71, 1.25)	0.88 (0.58, 1.30)	1.07 (0.82, 1.42)	1.04 (0.82, 1.32)	1.05 (0.97, 1.14)	1.10 (1.00, 1.21)
Forest	0.79 (0.60, 1.02)	0.74 (0.51, 1.03)	1.08 (0.62, 1.90)	0.86 (0.53, 1.36)	1.02 (0.92, 1.12)	0.98 (0.88, 1.10)

*OR* odds ratio, *UI* uncertainty interval

Cropland was positively associated with malaria at low levels of transmission intensity. Each 10-percentage point increase in cropland cover was associated with a crude malaria prevalence odds ratio (POR) of 2.00 (95% UI: 1.19, 3.47), which increased to 2.44 (95% UI 1.27, 5.11) when adjusting for confounders. Increasing cropland cover was not associated with malaria prevalence at moderate (POR: 0.87, 95% UI: 0.60, 1.24) or high (POR: 0.94, 95% UI: 0.84, 1.06) transmission intensity, when adjusting for confounders.

Grassland cover exhibited a small association with increased malaria prevalence at high levels of transmission intensity, although the 95% UIs included the null. When adjusting for confounding variables, each 10-percentage point increase in grassland cover was associated with a malaria prevalence odds ratio of 1.10 (95% UI: 1.00, 1.21). At moderate levels of transmission intensity, estimates were shifted toward the null (POR: 1.04, 95% UI: 0.82, 1.32), at low transmission intensity, estimates crossed the null, but remained close to 1 (POR: 0.88, 95% UI: 0.58, 1.30).

Forest cover showed a small association with decreased prevalence of malaria in low-transmission intensity areas; however, UIs included the null. Adjusted estimates indicated a malaria POR of 0.74 (0.51, 1.03) for each 10-percentage point increase in forest cover at low-transmission intensity. Estimates were shifted closer to the null with UIs spanning the null at moderate (POR: 0.86, 95% UI: 0.53, 1.36) and high (POR: 0.98, 95% UI: 0.88, 1.10) transmission intensity, adjusting for confounding variables.

## Discussion

In this study, using data from a nationally representative survey of school-aged children in Tanzania, higher cropland cover was found to be associated with increased malaria with a POR of 2.44 (95% UI: 1.27, 5.11) in low-transmission intensity settings but not at higher levels of transmission when controlling for confounders. Associations found between other land cover classes with malaria also tended to be dependent on background transmission intensity. Grassland cover exhibited a small association with increased malaria prevalence odds at higher-transmission intensity settings and conversely, forest cover had a small association with lower prevalence odds of malaria in low transmission intensity areas, however neither estimate was statistically significant.

Similar associations between land cover and malaria have been reported previously in Tanzania and neighbouring countries. Land use for agriculture has been found to be associated with increased transmission of malaria among children in Tanzania [8–10], DRC [6], Malawi [7], Kenya, and Burundi [9, 12], and increased abundance of *Anopheles* vectors [34, 35]. The present study did not collect data on crop types; however, previous studies in Tanzania have suggested irrigated rice fields confer elevated risk of malaria over pastoral land cover [27], or savannah [8]. A complementary study in Kenya found rice fields and nearby canals to be associated with *Anopheles* abundance and diversity, with higher larval densities in areas where homes were located close to rice fields [35]. Contrary to the current study results, Chacky et al. [16] found multiple types of forest cover (rainforest, dry, deciduous) to be associated with increased malaria prevalence relative to grassland among school-aged children. This study found that increasing grassland corresponded with a small increase in malaria prevalence at high transmission intensity, and it was also found that increasing forest cover may have conferred slight protection, although only at low levels of transmission intensity. This may be an artifact of reduced cropland, and thus lower malaria, in areas with increased forest. However, deforested areas, which are often cleared for cropland, have been associated with increased vectorial capacity of *Anopheles gambiae* and may translate to higher malaria risk relative to forested areas [36]. The results of this study however are not directly comparable with Chacky et al. due to different land cover/ecological zone classification methods and may have also differ due to the stratification of analyses by transmission intensity.

This study is the first analysis of land cover and malaria prevalence in Tanzania to stratify by transmission intensity that showed differing associations between land cover and malaria by transmission level. This finding has important implications for malaria control and policy in Tanzania, such as the need for continued evaluation of malaria risk factors by transmission intensity and distribution of malaria control interventions and resources accordingly. The importance of transmission stratification on land cover-malaria relationships is likely not unique to Tanzania and future studies should consider evaluating malaria risk factors by transmission intensity in other countries. The World Health Organization (WHO) recommends stratification of malaria burden to optimize distribution of malaria control interventions [28, 29]. Additionally, knowledge of how relationships between intervenable malaria risk factors and malaria may differ by transmission strata offers additional ways to finely target interventions and policies. The finding that increasing cropland is associated with increased malaria prevalence in areas of low transmission intensity provides a new target for further reducing the burden of malaria in low-transmission settings. At moderate- and high-transmission intensities, this study failed to detect significant associations between all land cover classes and malaria prevalence. It is possible that higher levels of community transmission prevented detection of strong relationships between different land cover classes and malaria given the spatial resolution of the data. As transmission intensity declines, patterns of malaria become increasing heterogenous and focal, allowing for characterization of geographic features associated with more intense transmission of malaria, which can further enhance malaria control efforts. In this context, it is unsurprising that strong relationships between land cover and malaria prevalence were not detected outside of low transmission areas. These results highlight the importance of strengthening current malaria control interventions at all levels of transmission, and in areas with low transmission, the use of complementary vector control interventions, such as environmental manipulation and larval source management, should additionally be considered around croplands and agricultural areas. As areas with low transmission push toward the goal of malaria elimination, it is increasingly important to assess for environmental risk factors to maximize detection of malaria risk patterns. Indeed, understanding the ecology of malaria transmission is necessary for optimizing interventions and is a pre-requisite to malaria elimination [37].

Current malaria control interventions in Tanzania centre on the use of insecticide-treated nets (ITNs) and indoor residual spraying, which aim to reduce malaria transmission within households [31]. Although these interventions are the backbone of malaria control across much of sub-Saharan Africa, they are facing increasing numbers of obstacles, including insecticide resistance, changes to vector species population dynamics, vector behavioural adaptations, and improper use leading to insufficient protection of household residents [37, 38]. In Tanzania, widespread use of ITNs and household spraying has led to a shift in the composition of vector species with *Anopheles arabiensis* replacing *An. gambiae *sensu stricto (*s.s.*) as the predominant vector in many areas [31, 39]. *Anopheles arabiensis* exhibits broader feeding and resting preferences compared to *An. gambiae s.s.*, allowing the species to evade individual and household-targeted interventions [39]. This gives rise to a new hypothesis that such changes in vector dynamics in areas of low transmission may have contributed to the strong association observed between higher cropland cover and increased malaria prevalence at low transmission intensities. Thus, to achieve wide-spread vector control and to suppress malaria transmission further, interventions outside of the house, such as larval source management, space spraying and other environmental manipulation and management techniques need to be explored further and implemented in areas with low levels of malaria transmission [38]. The identification of specific land cover or land use practices associated with malaria, such as cropland in this study, provides an actionable target for highly effective, yet under-utilized, malaria vector control interventions (i.e., space spraying, vector habitat management or manipulation, biological control, and larvicides) that could complement current malaria control activities and maximize progress toward malaria elimination.

This study has several important limitations. Primarily, there was limited data on malaria interventions such as bed net use or indoor residual spraying (IRS), or other important household level sociodemographic variables, such as wealth and housing materials. ITN use is high in Tanzania with an estimated 78% of households owning at least one net [40] and 68% of school-aged children reported sleeping under a net the previous night in 2015 [16]. Although the use of ITNs is high in Tanzania, distribution of malaria control interventions is based on sub-national classifications of transmission intensity.

While ownership of ITNs and use of IRS should be similar within each stratum of transmission and independent of local land cover dynamics, variation is likely to occur between levels of transmission and this possible source of bias was unable to be controlled for; caution should be exercised when comparing results between levels of transmission. Wealth or household construction were also unable to be controlled for, both of which are important risk factors for malaria [19, 41]. An attempt was made to control for population density, which is highly correlated with urbanicity and associated with higher average wealth and improved housing construction. Thus, little uncontrolled confounding bias due to wealth and/or housing construction is expected in the confounder-adjusted estimates. Secondly, the cross-sectional design of the SMPS precluded assessment of temporal trends and any changes in malaria prevalence due to seasonality. The SMPS was primarily administered at the start of the rainy season for each region, however at some locations it is possible that the timing of the survey was misaligned with the start of the rainy season; any bias from seasonality that was not captured by measures of temperature, precipitation, and vegetation in the analyses could have biased the results downward and toward the null. Additionally, the study sample was limited to children attending school at the selected school location in eight regions of Tanzania. School-based malaria surveys have been shown to strongly correlate with community malaria estimates [42], although absolute measures of disease burden may be higher in school surveys due to the higher age-associated risk of school-aged individuals. However, relative measures of risk, as were used in this study, should be unaffected. Further, school attendance in Tanzania is mandatory, thus the surveyed sample of students from randomly selected schools should be representative of the national population of school-aged children in Tanzania. Finally, the regions in this study were selected to be representative of varying strata of malaria risk across the country and the results are expected to be generalizable to the whole of mainland Tanzania during the 2017 study period.

Associations between land cover and malaria prevalence were found to be dependent on background levels of transmission intensity in Tanzania. In particular, in areas of low-transmission intensity, higher cropland cover was associated with increased malaria prevalence. Low-transmission intensity areas have high potential to achieve malaria elimination goals and expanding the current arsenal of malaria control interventions to include complementary vector control interventions in communities exposed to cropland or engaged in agricultural work may help reduce the burden of malaria further in these areas. As malaria transmission dynamics change in response to increasing interventions and transmission becomes increasingly stratified, it is important to re-evaluate how malaria risk factors may differ by strata of transmission intensity to improve prioritization of resources and continue making progress in malaria control.

## Supplementary Information

**Additional file 1: Table S1.** Prevalence estimates for each district council included in the study. **Table S2.** Estimated beta values and 95% uncertainty intervals for covariates included in adjusted models for land cover and log-odds of malaria prevalence, stratified by transmission intensity. **Table S3.** Posterior malaria odds ratio estimates for cropland, grassland, and forest land cover stratified by transmission intensity comparing adjusted, non-spatial model results and adjusted model results including a spatially varying intercept.

## Data Availability

The datasets generated and analysed during the present study are available from the corresponding author upon request.

## Abbreviations

CCI: Climate Change Initiative
CHIRPS: Climate Hazards Group InfraRed Precipitation with Station
DBS: Dried blood spots
DIC: Deviance information criterion
DRC: Democratic Republic of the Congo
EVI: Enhanced Vegetation Index
IQR: Interquartile range
ITN: Insecticide-treated net
IRS: Indoor residual spraying
POR: Prevalence odds ratio
SMPS: School Malaria Parasitological Survey
UI: Uncertainty interval
WHO: World Health Organization

## References

[1] Weiss DJ, Lucas TCD, Nguyen M, Nandi AK, Bisanzio D, Battle KE (2019). Mapping the global prevalence, incidence, and mortality of *Plasmodium falciparum*, 2000–17: a spatial and temporal modelling study. Lancet.

[2] Noor AM, Kinyoki DK, Mundia CW, Kabaria CW, Mutua JW, Alegana VA (2014). The changing risk of *Plasmodium falciparum* malaria infection in Africa: 2000–10: a spatial and temporal analysis of transmission intensity. Lancet.

[3] Snow RW, Sartorius B, Kyalo D, Maina J, Amratia P, Mundia CW (2017). The prevalence of *Plasmodium falciparum* in sub Saharan Africa since 1900. Nature.

[4] Weiss DJ, Mappin B, Dalrymple U, Bhatt S, Cameron E, Hay SI (2015). Re-examining environmental correlates of *Plasmodium falciparum* malaria endemicity: a data-intensive variable selection approach. Malar J.

[5] Griffin JT, Hollingsworth TD, Okell LC, Churcher TS, White M, Hinsley W (2010). Reducing *Plasmodium falciparum* malaria transmission in Africa: a model-based evaluation of intervention strategies. PLoS Med.

[6] Janko MM, Irish SR, Reich BJ, Peterson M, Doctor SM, Mwandagalirwa MK (2018). The links between agriculture, *Anopheles* mosquitoes, and malaria risk in children younger than 5 years in the Democratic Republic of the Congo: a population-based, cross-sectional, spatial study. Lancet Planet Health.

[7] Townes LR, Mwandama D, Mathanga DP, Wilson ML (2013). Elevated dry-season malaria prevalence associated with fine-scale spatial patterns of environmental risk: a case–control study of children in rural Malawi. Malar J.

[8] Rumisha SF, Shayo EH, Mboera LEG (2019). Spatio-temporal prevalence of malaria and anaemia in relation to agro-ecosystems in Mvomero district. Tanzania Malar J.

[9] Ijumba JN, Mosha FW, Lindsay SW (2002). Malaria transmission risk variations derived from different agricultural practices in an irrigated area of northern Tanzania. Med Vet Entomol.

[10] Mboera LEG, Bwana VM, Rumisha SF, Malima RC, Mlozi MRS, Mayala BK (2015). Malaria, anaemia and nutritional status among schoolchildren in relation to ecosystems, livelihoods and health systems in Kilosa District in central Tanzania. BMC Public Health.

[11] Ijumba JN, Lindsay SW (2001). Impact of irrigation on malaria in Africa: paddies paradox. Med Vet Entomol.

[12] Carnevale P, Guillet P, Robert V, Fontenille D, Doannio J, Coosemans M (1999). Diversity of malaria in rice growing areas of the Afrotropical region. Parassitologia.

[13] Curtis PG, Slay CM, Harris NL, Tyukavina A, Hansen MC (2018). Classifying drivers of global forest loss. Science.

[14] President’s Malaria Initiative Tanzania Malaria Operational Plan. 2015:95.

[15] Thawer SG, Chacky F, Runge M, Reaves E, Mandike R, Lazaro S (2020). Sub-national stratification of malaria risk in mainland Tanzania: a simplified assembly of survey and routine data. Malar J.

[16] Chacky F, Runge M, Rumisha SF, Machafuko P, Chaki P, Massaga JJ (2018). Nationwide school malaria parasitaemia survey in public primary schools, the United Republic of Tanzania. Malar J.

[17] Hagenlocher M, Castro MC (2015). Mapping malaria risk and vulnerability in the United Republic of Tanzania: a spatial explicit model. Popul Health Metr.

[18] Tanzania Economic Update. World Bank Group; 2019:1–84.

[19] Deutsch-Feldman M, Brazeau NF, Parr JB, Thwai KL, Muwonga J, Kashamuka M (2020). Spatial and epidemiological drivers of *Plasmodium falciparum* malaria among adults in the Democratic Republic of the Congo. BMJ Glob Health.

[20] Land Cover CCI Algorithm Theoretical Basis Document Version 2. UCL‐Geomatics; 2013.

[21] Kaufmann C, Briegel H (2004). Flight performance of the malaria vectors *Anopheles gambiae* and *Anopheles atroparvus*. J Vector Ecol.

[22] Paaijmans KP, Read AF, Thomas MB (2009). Understanding the link between malaria risk and climate. Proc Natl Acad Sci USA.

[23] Blanford JI, Blanford S, Crane RG, Mann ME, Paaijmans KP, Schreiber KV (2013). Implications of temperature variation for malaria parasite development across Africa. Sci Rep.

[24] Waring RH, Coops NC, Fan W, Nightingale JM (2006). MODIS enhanced vegetation index predicts tree species richness across forested ecoregions in the contiguous U.S.A.. Remote Sens Environ.

[25] Midekisa A, Senay G, Henebry GM, Semuniguse P, Wimberly MC (2012). Remote sensing-based time series models for malaria early warning in the highlands of Ethiopia. Malar J.

[26] Drakeley CJ, Carneiro I, Reyburn H, Malima R, Lusingu JPA, Cox J (2005). Altitude-dependent and -independent variations in *Plasmodium falciparum* prevalence in northeastern Tanzania. J Infect Dis.

[27] Runge M, Snow RW, Molteni F, Thawer S, Mohamed A, Mandike R (2020). Simulating the council-specific impact of anti-malaria interventions: a tool to support malaria strategic planning in Tanzania. PLoS ONE.

[28] WHO. World malaria report (2020). 20 years of global progress and challenges.

[29] WHO. Global technical strategy for malaria, 2016–2030. Geneva: World Health Organization; 2015.

[30] Bhatt S, Weiss DJ, Cameron E, Bisanzio D, Mappin B, Dalrymple U (2015). The effect of malaria control on *Plasmodium falciparum* in Africa between 2000 and 2015. Nature.

[31] National Malaria Control Programme, WHO, Ifakara Health Institute, the INFORM Project. An epidemiological profile of malaria and its control in mainland Tanzania. Report funded by Roll Back Malaria and Department for International Development-UK; 2013.

[32] Lindgren F, Rue H. Bayesian Spatial Modelling with *R*—INLA. J Stat Softw. 2015;63. http://www.jstatsoft.org/v63/i19/. Accessed Apr 2021.

[33] Spiegelhalter DJ, Best NG, Carlin BP, Linde AVD (2002). Bayesian measures of model complexity and fit. J R Stat Soc Ser B Stat Methodol.

[34] Arisco NJ, Rice BL, Tantely LM, Girod R, Emile GN, Randriamady HJ (2020). Variation in *Anopheles* distribution and predictors of malaria infection risk across regions of Madagascar. Malar J.

[35] Mwangangi JM, Shililu J, Muturi EJ, Muriu S, Jacob B, Kabiru EW (2010). *Anopheles* larval abundance and diversity in three rice agro-village complexes Mwea irrigation scheme, central Kenya. Malar J.

[36] Afrane YA, Little TJ, Lawson BW, Githeko AK, Yan G (2008). Deforestation and vectorial capacity of *Anopheles gambiae* Giles mosquitoes in malaria transmission. Kenya Emerg Infect Dis.

[37] Ferguson HM, Dornhaus A, Beeche A, Borgemeister C, Gottlieb M, Mulla MS (2010). Ecology: a prerequisite for malaria elimination and eradication. PLoS Med.

[38] Killeen GF, Tatarsky A, Diabate A, Chaccour CJ, Marshall JM, Okumu FO (2017). Developing an expanded vector control toolbox for malaria elimination. BMJ Glob Health.

[39] Russell TL, Govella NJ, Azizi S, Drakeley CJ, Kachur SP, Killeen GF (2011). Increased proportions of outdoor feeding among residual malaria vector populations following increased use of insecticide-treated nets in rural Tanzania. Malar J.

[40] Ministry of Health, Community Development, Gender, Elderly and Children (MoHCDGEC) [Tanzania Mainland], Ministry of Health (MoH) [Zanzibar], National Bureau of Statistics (NBS), Office of the ChiefGovernment Statistician (OCGS), ICF. Tanzania Malaria Indicator Survey 2017. Dar es Salaam, Tanzania, and Rockville, Maryland, USA: MoHCDGEC, MoH, NBS, OCGS, and ICF; 2017. https://dhsprogram.com/pubs/pdf/MIS31/MIS31.pdf. Accessed July 2021.

[41] Tusting LS, Bottomley C, Gibson H, Kleinschmidt I, Tatem AJ, Lindsay SW (2017). Housing improvements and malaria risk in Sub-Saharan Africa: a multi-country analysis of survey data. PLoS Med.

[42] Stevenson JC, Stresman GH, Gitonga CW, Gillig J, Owaga C, Marube E (2013). Reliability of school surveys in estimating geographic variation in malaria transmission in the western Kenyan highlands. PLoS ONE.

