# Supplementary material for: Evaluating malaria prevalence and land cover across varying transmission intensity in Tanzania using a cross-sectional survey of school-aged children
Source: Malar J. 2022 Mar 9;21:80. doi: 10.1186/s12936-022-04107-8 (PMC8905829; doi:10.1186/s12936-022-04107-8)
Supplement: Supplementary file 1 — Additional file 1: Table S1. Prevalence estimates for each district council included in the study. Table S2. Estimated beta values and 95% uncertainty intervals for covariates included in adjusted models for land cover and log-odds of malaria prevalence, stratified by transmission intensity. Table S3. Posterior malaria odds ratio estimates for cropland, grassland, and forest land cover stratified by transmission intensity comparing adjusted, non-spatial model results and adjusted model results including a spatially varying intercept. [file 12936_2022_4107_MOESM1_ESM.docx]

**Table S1**  **Prevalence estimates for each district council included in the study**

| Region | District council | Transmission intensity | Pupils (n) | *P. falciparum*-positive (n) | Prevalence (%) |
| --- | --- | --- | --- | --- | --- |
| Arusha | Arusha CC | low | 689 | 3 | 0.4 |
| Arusha | Arusha DC | low | 484 | 0 | 0 |
| Arusha | Karatu | low | 433 | 0 | 0 |
| Arusha | Longido | low | 256 | 0 | 0 |
| Arusha | Meru | low | 430 | 2 | 0.5 |
| Arusha | Monduli | low | 117 | 0 | 0 |
| Arusha | Ngorongoro | low | 348 | 0 | 0 |
| Iringa | Iringa DC | low | 338 | 3 | 0.9 |
| Iringa | Iringa MC | low | 314 | 0 | 0 |
| Iringa | Kilolo DC | low | 316 | 3 | 0.9 |
| Iringa | Mafinga TC | low | 130 | 0 | 0 |
| Iringa | Mufindi | low | 485 | 3 | 0.6 |
| Kagera | Biharamulo | high | 463 | 194 | 41.9 |
| Kagera | Bukoba DC | high | 180 | 73 | 40.6 |
| Kagera | Bukoba MC | low | 271 | 7 | 2.6 |
| Kagera | Karagwe | high | 332 | 77 | 23.2 |
| Kagera | Kyerwa | high | 392 | 115 | 29.3 |
| Kagera | Missenyi | high | 204 | 57 | 27.9 |
| Kagera | Muleba | high | 868 | 196 | 22.6 |
| Kagera | Ngara | high | 368 | 97 | 26.4 |
| Mara | Bunda DC | high | 293 | 83 | 28.3 |
| Mara | Bunda TC | moderate | 37 | 25 | 67.6 |
| Mara | Butiama | high | 280 | 73 | 26.1 |
| Mara | Musoma DC | high | 241 | 59 | 24.5 |
| Mara | Musoma MC | moderate | 274 | 7 | 2.6 |
| Mara | Rorya | high | 418 | 194 | 46.4 |
| Mara | Serengeti | high | 271 | 104 | 38.4 |
| Mara | Tarime DC | high | 317 | 148 | 46.7 |
| Mara | Tarime TC | moderate | 97 | 6 | 6.2 |
| Mtwara | Masasi DC | high | 382 | 120 | 31.4 |
| Mtwara | MasasiTC | high | 133 | 27 | 20.3 |
| Mtwara | Mtwara DC | high | 167 | 95 | 56.9 |
| Mtwara | Mtwara MC | high | 89 | 29 | 32.6 |
| Mtwara | Nanyamba TC | high | 121 | 49 | 40.5 |
| Mtwara | Nanyumbu | high | 256 | 91 | 35.5 |
| Mtwara | Newala DC | high | 68 | 56 | 82.4 |
| Mtwara | Tandahimba | high | 295 | 135 | 45.8 |
| Rukwa | Kalambo | moderate | 232 | 45 | 19.4 |
| Rukwa | Nkasi | moderate | 285 | 163 | 57.2 |
| Rukwa | Sumbawanga DC | low | 699 | 44 | 6.3 |
| Rukwa | Sumbawanga MC | low | 290 | 1 | 0.3 |
| Tabora | Igunga | moderate | 513 | 39 | 7.6 |
| Tabora | Kaliua | high | 368 | 160 | 43.5 |
| Tabora | Nzega DC | high | 345 | 125 | 36.2 |
| Tabora | Nzega TC | high | 75 | 14 | 18.7 |
| Tabora | Sikonge | high | 274 | 48 | 17.5 |
| Tabora | Tabora MC | high | 202 | 37 | 18.3 |
| Tabora | Urambo | high | 268 | 82 | 30.6 |
| Tanga | Bumbuli | low | 306 | 0 | 0 |
| Tanga | Handeni DC | high | 481 | 196 | 40.7 |
| Tanga | Handeni TC | high | 124 | 43 | 34.7 |
| Tanga | Kilindi | high | 311 | 52 | 16.7 |
| Tanga | Korogwe DC | moderate | 198 | 8 | 4 |
| Tanga | Korogwe TC | moderate | 61 | 1 | 1.6 |
| Tanga | Lushoto DC | low | 239 | 3 | 1.3 |
| Tanga | Mkinga | moderate | 144 | 21 | 14.6 |
| Tanga | Muheza DC | high | 220 | 39 | 17.7 |
| Tanga | Pangani | moderate | 60 | 0 | 0 |
| Tanga | Tanga CC | moderate | 279 | 36 | 12.9 |

**Table S2 Estimated beta values and 95% uncertainty intervals for covariates included in adjusted models for land cover and log-odds of malaria prevalence, stratified by transmission intensity**

|  | **Low** | | **Moderate** | | **High** | |
| --- | --- | --- | --- | --- | --- | --- |
|  | **β** | **95% UI** | **β** | **95% UI** | **β** | **95% UI** |
| **Cropland** |  |  |  |  |  |  |
| Age | 0.266 | 0.009, 0.530 | 0.168 | 0.032, 0.305 | 0.193 | 0.142, 0.244 |
| Gender | -0.122 | -0.633, 0.386 | 0.183 | -0.091, 0.458 | 0.316 | 0.219, 0.413 |
| Temperature | -0.135 | -1.442, 1.183 | -0.905 | -2.164, 0.44 | -0.061 | -0.445, 0.320 |
| Precipitation | -0.062 | -1.048, 0.792 | 1.824 | -0.555, 4.204 | 0.064 | -0.261, 0.389 |
| Vegetation | 0.000 | -1.544, 1.587 | -0.776 | -1.711, 0.213 | -0.219 | -0.576, 0.135 |
| Elevation | -1.074 | -2.233, -0.029 | -0.759 | -1.738, 0.194 | -0.174 | -0.567, 0.219 |
| Population density | 0.092 | -0.426, 0.608 | -2.672 | -4.147, -1.341 | -1.088 | -2.095, -0.106 |
| Proximity to water body | 0.655 | -0.036, 1.431 | 1.187 | 0.572, 1.859 | 0.010 | -0.211, 0.231 |
| **Grassland** |  |  |  |  |  |  |
| Age | 0.267 | 0.010, 0.531 | 0.168 | 0.032, 0.305 | 0.192 | 0.141, 0.244 |
| Gender | -0.122 | -0.634, 0.386 | 0.183 | -0.091, 0.458 | 0.316 | 0.219, 0.413 |
| Temperature | 0.313 | -1.243, 1.996 | -1.151 | -2.231, -0.003 | -0.221 | -0.649, 0.201 |
| Precipitation | -0.369 | -1.618, 0.687 | 1.642 | -0.714, 3.978 | 0.143 | -0.178, 0.466 |
| Vegetation | 0.294 | -1.388, 2.084 | -0.882 | -1.839, 0.114 | -0.32 | -0.696, 0.051 |
| Elevation | -0.93 | -2.301, 0.322 | -0.704 | -1.771, 0.349 | -0.264 | -0.643, 0.114 |
| Population density | -0.099 | -0.667, 0.446 | -2.794 | -4.269, -1.453 | -1.353 | -2.411, -0.329 |
| Proximity to water body | 0.457 | -0.290, 1.300 | 1.170 | 0.551, 1.842 | -0.027 | -0.247, 0.194 |
| **Forest** |  |  |  |  |  |  |
| Age | 0.265 | 0.009, 0.529 | 0.168 | 0.032, 0.305 | 0.193 | 0.142, 0.244 |
| Gender | -0.122 | -0.633, 0.385 | 0.183 | -0.091, 0.458 | 0.316 | 0.218, 0.413 |
| Temperature | -0.028 | -1.367, 1.307 | -1.236 | -2.313, -0.098 | -0.036 | -0.418, 0.343 |
| Precipitation | -0.290 | -1.322, 0.606 | 1.541 | -0.768, 3.828 | 0.087 | -0.235, 0.410 |
| Vegetation | 0.410 | -1.146, 2.015 | -0.797 | -1.724, 0.169 | -0.180 | -0.537, 0.176 |
| Elevation | -1.015 | -2.244, 0.065 | -0.604 | -1.488, 0.274 | -0.232 | -0.617, 0.153 |
| Population density | -0.005 | -0.545, 0.522 | -2.970 | -4.515, -1.56 | -1.092 | -2.142, -0.067 |
| Proximity to water body | 0.452 | -0.235, 1.223 | 1.159 | 0.553, 1.820 | 0.003 | -0.219, 0.225 |

**Table S3 Posterior malaria odds ratio estimates for cropland, grassland, and forest land cover stratified by transmission intensity comparing adjusted, non-spatial model results and adjusted model results including a spatially varying intercept**.

|  | **Low** | | **Moderate** | | **High** | |
| --- | --- | --- | --- | --- | --- | --- |
|  | Odds ratio (95% UI) | DIC | Odds ratio (95% UI) | DIC | Odds ratio (95% UI) | DIC |
| **Cropland** |  |  |  |  |  |  |
| non-spatial | 2.44 (1.27, 5.11) | 573.3001 | 0.87 (0.60, 1.24) | 1379.195 | 0.94 (0.84, 1.06) | 9,755.739 |
| spatial | 1.06 (0.36, 2.85) | 575.3788 | 1.09 (0.78, 1.51) | 1378.682 | 0.95 (0.83, 1.08) | 9,755.554 |
| **Forest** |  |  |  |  |  |  |
| non-spatial | 0.74 (0.51, 1.03) | 575.4052 | 0.86 (0.53, 1.36) | 1,379.604 | 0.98 (0.88, 1.10) | 9,756.062 |
| spatial | 0.98 (0.64, 1.48) | 575.3767 | 1.03 (0.69, 1.54) | 1,378.811 | 0.96 (0.84, 1.09) | 9,755.644 |
| **Grassland** |  |  |  |  |  |  |
| non-spatial | 0.88 (0.58, 1.30) | 576.1789 | 1.04 (0.82, 1.32) | 1,379.628 | 1.10 (1.00, 1.21) | 9,755.282 |
| spatial | 0.95 (0.65, 1.33) | 575.1922 | 0.99 (0.79, 1.22) | 1,378.872 | 1.10 (0.99, 1.21) | 9,755.123 |

UI: Uncertainty interval; DIC: Deviance information criterion.
